# The *SLC2A9 *nonsynonymous Arg265His variant and gout: evidence for a population-specific effect on severity

**DOI:** 10.1186/ar3356

**Published:** 2011-06-09

**Authors:** Jade E Hollis-Moffatt, Peter J Gow, Andrew A Harrison, John Highton, Peter BB Jones, Lisa K Stamp, Nicola Dalbeth, Tony R Merriman

**Affiliations:** 1 Department of Biochemistry, University of Otago, 710 Cumberland Street, Dunedin 9012, New Zealand; 2 Department of Rheumatology, Middlemore Hospital, 100 Hospital Road, Auckland 2025, New Zealand; 3 Department of Medicine, University of Otago, 23A Mein Street, Wellington 6242, New Zealand; 4 Department of Medicine, University of Otago, 201 Great King Street, Dunedin 9016, New Zealand; 5 Department of Medicine, University of Auckland, 2 Park Road, Auckland 1023, New Zealand; 6 Department of Medicine, University of Otago, 2 Riccarton Avenue, Christchurch 8140, New Zealand

## Abstract

**Introduction:**

The C allele of the nonsynonymous Arg265His (*rs3733591*) variant of *SLC2A9 *confers risk for gout in Han Chinese, Solomon Island and Japanese samples, with a stronger role in tophaceous gout. There is no evidence for an association with gout in Caucasian populations. In the present study, we tested *rs3733591 *for association with gout in New Zealand (NZ) Māori, Pacific Island and Caucasian samples.

**Methods:**

*Rs3733591 *was genotyped across gout patients (*n *= 229, 232 and 327 NZ Māori, Pacific Island and Caucasian samples, respectively) and non-gout controls (*n *= 343, 174 and 638 Māori, Pacific Island and Caucasian samples, respectively). Further Caucasian sample sets consisting of 67 cases and 4,712 controls as well as 153 cases and 6,969 controls were obtained from the Framingham Heart Study and the Atherosclerosis Risk in Communities study, respectively. The Polynesian samples were analyzed according to Eastern and Western Polynesian ancestry.

**Results:**

No evidence for risk conferred by the C allele of *rs3733591 *with gout was found in the sample sets of NZ Māori (odd ratio (OR) = 0.98, *P *= 0.86), Eastern Polynesians (OR = 0.99, *P *= 0.92), Western Polynesians (OR = 1.16, *P *= 0.36) or combined Caucasians (OR = 1.15, *P *= 0.13). The C allele was significantly overrepresented in Māori tophaceous cases compared to cases without tophi (OR = 2.21, *P *= 0.008), but not in the other ancestral groupings.

**Conclusions:**

Noting that our study's power was limited for detecting weak genetic effects, we were unable to replicate associations of *rs3733591 *with gout in Eastern Polynesian, Western Polynesian and Caucasian samples. However, consistent with a previous study of Han Chinese and Solomon Island populations, our data suggest that *rs3733591 *could be a marker of severe gout in some populations. Our results also suggest that the effect of this variant is population-specific, further confirming population heterogeneity regarding the association of *SLC2A9 *with gout.

## Introduction

Gout is a common form of inflammatory arthritis predominantly affecting men, with hyperuricemia being an essential predeterminant. As urate concentrations reach saturation in the blood, monosodium urate (MSU) crystals are deposited in the joints and tissues. An acute self-limiting inflammatory reaction to these MSU crystals leads to severe pain and debilitation (gout). Without resolution, the MSU crystals and subsequent inflammation can lead to chronic tophaceous gout, bony erosions and permanent disability. In New Zealand (NZ; Aotearoa), gout is common in Māori and Pacific Island men, with the prevalence estimated to range from 9.3% to 13.9% and 14.9%, respectively [1,2]. Renal underexcretion of uric acid has been determined to be an underlying characteristic of gout and is more pronounced in people of Māori and Pacific Island descent, and even more so in patients with hyperuricemia and/or gout [3,4].

Genomewide association studies in Caucasian cohorts have shown that intronic variants (*rs7442295 *and surrogate marker *rs11942223*) within the solute carrier family 2, member 9/facilitated glucose transporter 9 (*SLC2A9/GLUT9*) gene are associated with high serum urate concentrations and gout [5-10]. The intronic *SLC2A9 *variant *rs11942223 *best explains the strong role that *SLC2A9 *played in the development of gout in NZ Māori, Pacific Island and Caucasian sample sets [11]. Interestingly, this variant is very rare in Chinese, Japanese and Solomon Island people and does not play a genetic role in the development of gout in these populations [12,13]. The intronic *SLC2A9 *variants have gender-specific effects on serum urate, with the effect being stronger in women [14].

SLC2A9 has been confirmed to be a renal urate transporter [9,15,16]. It is a urate reuptake molecule that has two isoforms, with the long-form being expressed on the basal side and the short-form being expressed on the apical side of the proximal renal tubule [17]. Mice with overexpression of the long-form of SLC2A9 (also known as hURATv1) on the basolateral surface have a much greater reuptake of urate from the lumen into the blood and reduced urinary urate excretion [15]. In contrast, overexpression of the URAT1 renal urate transporter does not enhance urate reabsorption, indicating that SLC2A9 is the rate-limiting step in urate reuptake in mice [17].

An additional *SLC2A9 *variant, R265H (*rs3733591*), contributes significantly to the development of elevated urate concentrations and gout in Han Chinese, Solomon Island and Japanese sample sets [12,13], but not in a Caucasian sample set [8]. Han Chinese and Solomon Island gout patients with the risk C allele had a higher risk for tophi [12]. There are currently no data indicating how R265H may influence SLC2A9 function. Importantly, the effect of R265H in these populations is independent of the previously gout-associated intronic *rs7442295/rs11942223 *variants. The measure of linkage disequilibrium, *r*^2^, is < 0.05 between *rs3733591 *and *rs11942223 *in HapMap Caucasian, Chinese and Japanese samples. Given the existing evidence for population heterogeneity in association of *SLC2A9 *variants (intronic and R265H) with gout [8,11-13], we investigated a possible role for R265H (*rs3733591*) in gout in NZ Māori, Pacific Island and Caucasian case-control sample sets in a study adequately powered to detect an effect equivalent to that observed in other populations (odds ratio (OR) > 1.4).

## Material and methods

### Study participants

Genotyping of NZ sample sets of Māori (229 cases and 343 controls), Pacific Island (232 cases and 174 controls) and Caucasian (327 cases and 638 controls) was done (Table 1). All cases were recruited from rheumatology outpatient clinics, and their gout diagnoses were confirmed by a rheumatologist according to the American College of Rheumatology preliminary diagnostic criteria for acute gout [18]. The controls had no history of arthritis and were recruited from the wider community. Our recruitment of gout patients was approved by the NZ Multi-region Ethics Committee (MREC 05/10/130), and the recruitment of the controls was approved by the Lower South and Multi-region Ethics Committees (OTA/99/11/098 and MREC 05/10/130). All participants provided written informed consent for the collection of samples and subsequent analysis.

**Table 1:** Demographic and clinical characteristics of gout cases and controls^a^

	Gout						Controls					
Characteristics	Māori^b^	EP^b^	WP	NZ Caucasian	FHS	ARIC	Māori	NZ EP	NZ WP	NZ Caucasian	FHS	ARIC
Number of cases/controls	229	261	187	327	67	153	343	348	142	638	47,12	6,969
Males, %	78	76	95	85	94	73	31	33	57	42	45	45
Mean ± 1 SD grandparents of stated ancestry	2.87 ± 1.15	3.10 ± 1.07	3.72 ± 0.59	4.00 ± 0.00	-	-	2.33 ± 1.20	2.46 ± 1.22	3.49 ± 0.86	4.00 ± 0.00	-	-
Mean serum urate at recruitment, mmol/L (range)	0.45 (0.21 to 0.98)	0.47 (0.19 to 0.98)	0.49 (0.17 to 0.72)	0.41 (0.18 to 1.07)	0.42 (0.21 to 0.58)	0.42 (0.11-0.70)	-	-	-	-	0.29 (0.07 to 0.63)	0.34 (0.03 to 0.72)
Confirmed tophi, %	38	39	56	30	-	-	-	-	-	-	-	-
Mean age at onset, years (range)	39 (15 to 74)	39 (15 to 74)	34 (15 to 75)	47 (15 to 83)	-	-	-	-	-	-	-	-
Mean age at recruitment, years (range)	-	-	-	-	38 (18 to 55)	54 (45-65)	44 (17 to 80)	44 (17 to 85)	38 (17 to 86)	52 (17 to 95)	37 (5 to 72)	54 (44 to 66)
Mean gout attacks in past year, *n *(range)	11 (0 to > 1/week)	11 (0 to > 1/week)	16 (0 to > 1/week)	7.5 (0 to > 1/week)	-	-	-	-	-	-	-	-
First-degree relative with gout, %	63	64	53	42	-	-	30	31	23	14	-	-
Allopurinol treatment, %	83	82	87	78	-	-	-	-	-	-	-	-
Probenecid treatment, %	6	6	13	6	-	-	-	-	-	-	-	-
Mean BMI (range)	35 (22 to 62)	35 (22 to 66)	38 (22 to 93)	30 (19 to 62)	29 (20 to 37)	28 (21-41)	32 (20 to 77)	32 (20 to 77)	35 (21 to 63)	28 (19 to 56)	27 (15 to 61)	27 (15 to 56)
Type 2 DM, %	28	30	18	13	15	7	10	9	11	7	4	7
Hypertension, %	63	63	46	49	-	-	16	16	17	14	-	-
Dyslipidemia, %	51	52	53	48	-	-	12	12	11	16	-	-
Cardiovascular disease, %	40	38	20	37	-	-	3	3	1	6	-	-
Renal disease, %	30	30	22	22	21	-	2	2	1	2	6	-

^a^ARIC = Atherosclerosis Risk in Communities study; BMI = body mass index; DM = diabetes mellitus; EP = Eastern Polynesian; FHS = Framingham Heart Study; NZ = New Zealand; SD = standard deviation; WP = Western Polynesian. ^b^Full clinical data were available in 76% of Māori and 63% of EP gout cases (> 90% in other sample sets).

Given data from previous work investigating the *ABCG2 rs2231142 *variant in the NZ sample sets [19], along with knowledge of ancestral Polynesian and Māori migration [20-22], the groups analyzed were Māori and Eastern Polynesian (Māori and Cook Island), Western Polynesian (Tonga, Samoa, Niue and Tokelau) and Caucasian. A total of 223 cases and 327 controls overlapped between the Māori and Eastern Polynesian sample sets. People of mixed Eastern and Western Polynesian ancestry were excluded from the Eastern and Western Polynesian sample sets.

Fifty-five gout cases were obtained from the Framingham Heart Study (FHS) Offspring data set and combined with 17 gout cases from the Generation 3 data. Self-reported gout cases from the Offspring data set were included if the participants reported having gout on two or more survey occasions or reported having gout on one survey occasion and were also taking antigout medication. Self-reported gout cases from the Generation 3 data set were included if the participants had also answered "no" to taking medication for hypertension or high blood pressure. Control participants included those who were Caucasian and were unrelated to the gout patients. Genotypes were available for 67 gout cases and 4,712 control samples. One hundred fifty-three self-reported Caucasian gout patients who were not taking hypertensive medication were obtained from the Atherosclerosis Risk in Communities (ARIC) study and were compared to 6,969 unrelated controls.

### Genotyping

The *rs3733591 *variant of *SLC2A9 *was genotyped across the NZ Caucasian, Māori and Pacific Island sample sets using the TaqMan Allelic Discrimination Assay Kit (probe ID C__25803684_10; Applied Biosystems, Foster City, CA, USA) and a LightCycler 480 Real-Time Polymerase Chain Reaction System (Roche, Indianapolis, IN, USA).

### Statistical analysis

*A priori *calculations were performed to test the power of the NZ Polynesian sample sets to detect association of *rs3733591 *with gout on the basis of previous data [12,13]. Statistical power in the Māori, Eastern Polynesian and Western Polynesian sample sets was 79%, 82% and 58%, respectively (OR = 1.41, minor allele frequency = 0.346).

Allelic and genotypic frequencies were compared between case and control samples, and ORs and adherence to the Hardy-Weinberg equilibrium were calculated using the SHEsis package [23]. The genotype frequencies for *rs3733591 *were in Hardy-Weinberg equilibrium (*P *> 0.01) for all case and control sample sets.

Twenty-five biallelic markers were used as genomic controls to account for differing levels of non-Māori and non-Eastern Polynesian and non-Western Polynesian ancestry between the case and control samples. The stratification markers used were as follows: *rs2075876 (AIRE)*, *rs1816532 (ERBB4)*, *rs13419122 (GFPT1)*, *rs12401573 (SEMA4A)*, *rs6945435 (MGC87315)*, *rs743777 (IL2RB)*, *rs10511216 *(Intergenic), *rs12745968 (FAM69A)*, *rs1539438 (AP4B1)*, *rs729749 (NCF4)*, *rs3738919 (ITGAV)*, *rs1130214 (AKT1)*, *rs755622 (MIF)*, *rs7901695 (TCF7L2)*, *rs7578597 (THADA)*, *rs2043211 (CARD8)*, *rs10733113 (NLRP3)*, *rs900865 (SOX6)*, *rs2059606 (PGDS)*, *rs4129148 *(PseudoY), *rs831628 (CD59)*, *rs7725 (GFPT)*, *rs573816 *(upstream of *ALDOB)*, *rs1929480 (ALDOB) *and *rs12917707 (UMOD)*. There was an average allele frequency difference of 0.22 (0.03 to 0.61) between a subset of 469 Māori cases and controls and 505 Caucasian controls and a difference of 0.22 (allele frequency range 0.03 to 0.59) and 0.29 (0.04 to 0.67) between subsets of 417 Eastern Polynesian and 215 Western Polynesian cases and controls and 505 Caucasian controls, respectively. The genotype frequencies for the stratification markers all exhibited Hardy-Weinberg equilibrium *P *values > 0.003 for all case and control sample sets. STRUCTURE [24] was used to assign Māori, Eastern Polynesian and Western Polynesian individuals into non-Caucasian populations (parameters: number of populations was assumed to be two, 30,000 burn-in period and 1 million Markov chain Monte Carlo replications after burn-in). The 505 Caucasian control individuals were included in the STRUCTURE procedure to aid in population clustering as being representative of the ancestral Caucasian population. After running STRUCTURE on the Māori samples, the proportions of samples in the inferred Caucasian cluster were 0.95 for the 505 Caucasian controls and 0.06 for the total 572 Māori samples, 0.95 and 0.06 for the 608 Eastern Polynesian samples and 0.98 and 0.05 for the 330 Western Polynesian samples. The STRUCTURE output was used to run STRAT [24] to test for association (*P*_STRAT_) of the variant with disease in the presence of admixture. The phenotype of the 505 Caucasian individuals was set as unknown.

Gender and gender-genotype interaction analysis was performed using Stata software (StataCorp, College Station, TX, USA). Meta-analysis was performed to combine data from independent data sets using rmeta software (http://cran.r-project.org/web/packages/rmeta/index.html) (within Stata) to calculate the combined Mantel-Haenszel OR using a fixed effects model and the Breslow-Day test for heterogeneity between studies. Imputation of *rs3733591 *genotypes in the FHS and ARIC samples was done with IMPUTE2 using HapMap3 CEU (NCBI Build 36, db126b) as reference data and a quality threshold of 0.9.

## Results

**Figure 1:**
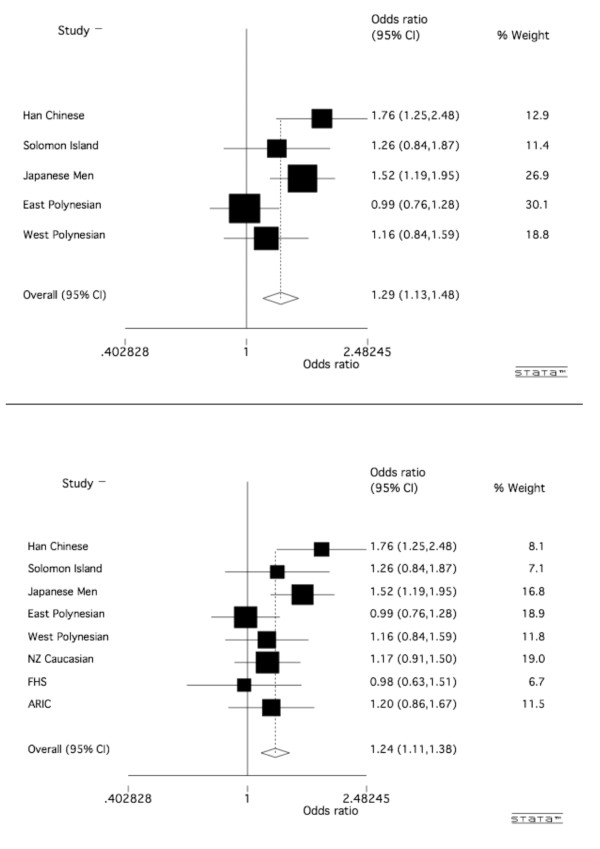
**Meta-analysis of association of *rs3733591 *with gout in Asian-Pacific ancestral groups without (top) and with (bottom) New Zealand (NZ) Caucasians**. ARIC = Atherosclerosis Risk in Communities study; CI = confidence interval; FHS = Framingham Heart Study.

The demographic and clinical characteristics of study participants are presented in Table 1 and genotype and allele distributions of the rs3733591 variant are shown in Table 2. There was no evidence for an association of the risk C allele of *rs3733591 *with gout in any of the Māori, Eastern Polynesian, Western Polynesian or Caucasian analyses (OR = 0.98, *P*_STRAT _= 0.93; OR = 0.99, *P*_STRAT _= 0.80; OR = 1.16, *P*_STRAT _= 0.65; and OR = 1.15, *P*_Meta _= 0.13, *P*_Breslow-Day _= 0.84, respectively). The *r*^2 ^values (measure of linkage disequilibrium) between the previously associated *SLC2A9 *variant, *rs11942223 *[11], and the variant tested here, *rs3733591*, were 0.04, 0.03, 0.04 and 0.05 for the Māori, Eastern Polynesian, Western Polynesian and Caucasian sample sets, respectively. Similarly, there was no evidence for an association on the basis of meta-analysis of the Eastern and Western Polynesian sample sets (OR = 1.05 (95% CI: 0.86 to 1.29); *P *= 0.62 and *P*_Breslow-Day _= 0.44, respectively). Meta-analysis of data from Polynesian, Han Chinese, Solomon Islands and Japanese sample sets (Table 2) [12,13] indicated heterogeneity (*P*_Breslow-Day _= 0.05); however, there was strong evidence for an association of *rs3733591 *in these combined Asian-Pacific populations (Figure 1) (OR = 1.29 (95% CI: 1.13 to 1.48, *P *= 1.6 × 10^-4^). Addition of Caucasian data to the meta-analysis weakened the overall effect; however, a strong association was maintained (Figure 1) (OR = 1.24 (95% CI: 1.13 to 1.48); P_Breslow-Day _= 0.13 and *P *= 1.1 × 10^-4^).

**Table 2:** Association analysis of *rs3733591 *with gout in NZ Māori, EP, WP and Caucasian sample sets and in the FHS cohort^a^

	Case genotypes^b^, *n *(OR)				Control genotypes, *n *(OR)							
Sample set	CC	CT	TT	C-allele frequency	CC	CT	TT	C-allele frequency	Genotypic *P*	Allelic *P*	*P*_STRAT_	Allelic OR, C allele (95% CI)
Māori	101 (0.500)	83 (0.411)	18 (0.089)	0.705	164 (0.500)	138 (0.421)	26 (0.079)	0.710	0.92	0.86	0.93	0.98 (0.74 to 1.28)
EP	119 (0.513)	92 (0.397)	21 (0.091)	0.711	169 (0.506)	139 (0.416)	26 (0.078)	0.714	0.82	0.92	0.80	0.99 (0.76 to 1.28)
WP	39 (0.227)	88 (0.512)	45 (0.262)	0.483	33 (0.239)	57 (0.413)	48 (0.348)	0.446	0.17	0.36	0.65	1.16 (0.84 to 1.59)
NZ Caucasian	217 (0.693)	87 (0.278)	9 (0.029)	0.832	417 (0.656)	196 (0.308)	23 (0.036)	0.810	0.49	0.23	-	1.17 (0.91 to 1.50)
FHS	44 (0.657)	21 (0.313)	2 (0.030)	0.813	3158 (0.670)	1,385 (0.294)	169 (0.036)	0.817	0.92	0.91	-	0.98 (0.63 to 1.51)
ARIC	117 (0.765)	30 (0.196)	6 (0.039)	0.863	4902 (0.704)	1,898 (0.272)	168 (0.024)	0.840	0.07	0.28	-	1.20 (0.86 to 1.67)

^a^ARIC = Atherosclerosis Risk in Communities study; CI = confidence interval; FHS = Framingham Heart Study; OR = odds ratio; EP = Eastern Polynesian; NZ = New Zealand; WP = Western Polynesian *P*_STRAT _= STRAT test for association. ^b^There was successful genotyping of 202 Māori cases (88%) and 328 Māori controls (96%), 232 EP cases (89%) and 334 EP controls (96%), 172 WP cases (92%) and 138 WP controls (97%) and 313 NZ Caucasian cases (96%) and 636 NZ Caucasian controls (100%).

A stronger effect of R265H with tophaceous gout has been reported [12]; therefore, we tested whether the effect of this variant is stronger in patients with tophi than in patients without tophi. We found significant overrepresentation of the C allele of *rs3733591 *in the group with tophaceous gout in the Māori analysis (OR = 2.21, *P*_STRAT _= 0.01), but not in the analyses of Eastern Polynesian (OR = 1.53, *P*_STRAT _= 0.17), Western Polynesian (OR = 0.97, *P*_STRAT _= 0.54) or NZ Caucasian (OR = 0.80, *P *= 0.33) population samples (Table 3).

**Table 3:** *Rs3733591 *and tophaceous gout in the Māori, EP, WP and NZ Caucasian cases^a^

	Tophaceous cases, *n *(OR)				Nontophaceous cases, *n *(OR)							
Sample set	CC	CT	TT	C-allele frequency	CC	CT	TT	C-allele frequency	Genotypic *P*	Allelic *P*	*P*_STRAT_	Allelic OR, C allele (95% CI)
Māori	36 (0.692)	13 (0.250)	3 (0.058)	0.817	37 (0.446)	37 (0.446)	9 (0.108)	0.669	0.02	0.008	0.01	2.21 (1.22 to 4.01)
EP	43 (0.652)	17 (0.258)	6 (0.091)	0.780	48 (0.490)	41 (0.418)	9 (0.092)	0.699	0.09	0.10	0.17	1.53 (0.92 to 2.55)
WP	22 (0.234)	46 (0.489)	26 (0.277)	0.479	16 (0.213)	41 (0.547)	18 (0.240)	0.487	0.76	0.88	0.54	0.97 (0.63 to 1.48)
NZ Caucasian	63 (0.656)	30 (0.313)	3 (0.031)	0.812	158 (0.715)	57 (0.258)	6 (0.027)	0.844	0.58	0.33	-	0.88 (0.51 to 1.24)

^a^CI, confidence interval; OR, odds ratio; CI, confidence interval; EP, Eastern Polynesian; NZ, New Zealand; WP, Western Polynesian; *P*_STRAT _= STRAT test for association.

Although the intronic *SLC2A9 *polymorphisms (*rs7442295/rs11942223*) have shown gender-specific effects on serum urate [14], there was no evidence for *rs3733591 *exerting a gender influence in the gout sample sets comprising NZ Māori (C/C, C/T, T/T genotypes in men and women were 78, 58 and 13 in men and 19, 18 and 3 in women; *P *= 0.78), Eastern Polynesians (C/C, C/T, T/T genotypes were 91, 61 and 15 in men and 24, 23 and 4 in women; *P *= 0.55) or NZ Caucasians (C/C, C/T, T/T genotypes in men and women were 187, 74 and 8 in men and 31, 13 and 1 in women; *P *= 0.95) (Western Polynesian samples were not stratified according to gender, as there were too few women (*n *= 6)). On the basis of logistic regression models, there was no evidence of an interaction between *rs3733591 *genotype and gender for the Māori, Eastern Polynesian or Caucasian sample sets (*P *= 0.77, *P *= 0.46 and *P *= 0.49, respectively). There was also no evidence for an association of *rs3733591 *with gout in the Māori, Eastern Polynesian or Caucasian sample sets when only males were analyzed (*P *= 0.59, *P *= 0.37 and *P *= 0.19, respectively).

## Discussion

The R265H nonsynonymous *SLC2A9 *variant has previously been demonstrated to be associated with gout and tophaceous gout in Han Chinese and Solomon Islanders [12], and it is associated with the development of gout in Japanese males [13]. We found no evidence for an association of this variant with gout in Māori, Eastern Polynesian, Western Polynesian or Caucasian sample sets (Table 2). However, consistent with the data from Han Chinese and Solomon Island sample sets [12], we found that the C allele conferred an increased risk of tophaceous gout in the Māori samples (OR = 2.21, *P*_STRAT _= 0.01), but not in the other sample sets (Table 3). At this stage, the simplest conclusion is that the C allele of *rs3733591 *has a weaker effect on gout *per se *in Caucasian and Polynesian populations than in the Asian and Melanesian populations studied thus far. However, it is very important to note that our sample sets had insufficient power to detect an association at OR < 1.4, although *a priori *power calculations indicated that they were sufficiently powered to detect an effect of OR > 1.4, which is equivalent to the risk observed in the sample sets described by Tu *et al*. [12] and Urano *et al*. [13]. Note that *post priori *power calculations for Eastern and Western Polynesian sample sets using the actual risk C-allele frequencies revealed similar power for OR = 1.4 (78% and 57%, respectively).

Despite not finding any evidence for an association of *rs3733591 *with gout in the Māori sample set (OR = 0.98), there was an effect conferred by the C allele when the Māori cases were stratified for the presence or absence of tophi (OR = 2.21). This supports the previous findings of Tu *et al*. [12], who concluded that *rs3733591 *might be a genetic checkpoint for tophaceous gout. Whether this is true will require further study in larger sample sets drawn from diverse ancestral groups. In the Asia-Pacific region, an effect for *rs3733591 *in tophaceous gout has been observed in the Han Chinese, Solomon Island and Polynesian NZ Māori populations, but not in Western Polynesian populations (Samoa, Tonga, Niue). Why this is the case is unclear; however, it is worth pointing out that unexpected heterogeneity in genetic association with gout in the Asia-Pacific region has also been documented at *ABCG2 *[19]. Furthermore, given that the C-allele frequency was similar between Māori and Caucasian sample sets (0.71 and 0.81, respectively, in controls), the disparate ORs for tophaceous gout are notable (OR = 2.21 and OR = 0.88, respectively, for tophaceous gout compared to nontophaceous gout). The reason for this is unclear; it could be a consequence of moderate sample size (power) or it could reflect differences in disease pathogenesis between the different population groups.

It is of interest that tophus formation is not consistently present in all patients with long-standing hyperuricemia and gout, suggesting that additional factors may regulate the development of these lesions. It is possible that genetic variation in *SLC2A9 *is one of these factors. SLC2A9 is expressed in the chondrocytes of human articular cartilage [25]. The minor allele of R265H (*rs3733591*, or one in linkage disequilibrium) may influence the activity of SLC2A9 in articular chondrocytes and increase the risk for deposition of MSU crystals and formation of tophi in joint structures. A further issue is that host factors other than hyperuricemia may contribute to the development of tophi; quantitative analysis of tophus histology indicates that, in contrast to the innate immune responses that are activated in acute gout, both innate and adaptive immunity are implicated in the development of the tophus [26]. Thus different immune responses to MSU crystals may lead to different manifestations of disease.

Interestingly, the C-allele risk variant of *rs3733591 *is the minor allele in control sample sets of Han Chinese (controls = 0.32) [12] Solomon Islanders (controls = 0.40) [12], Japanese (controls = 0.32) [13] and Western Polynesians (controls = 0.43), yet it is the major allele in sample sets taken from the FHS (controls = 0.82), NZ Caucasians (controls = 0.81) and Eastern Polynesians (controls = 0.71) (Table 2). There is a large shift in the minor allele frequency between Eastern and Western Polynesian sample sets, and such a change in allele frequency is consistent with that seen at another gout locus, *ABCG2 *(*rs2231142*) (the risk allele frequency is 0.29 in Western Polynesians, 0.13 in Caucasians and 0.10 in Eastern Polynesians) [19]. It can be observed that, as the frequency of the *rs3733591 *C allele increases, the risk conferred by *rs3733591 *for gout decreases, suggesting that population allele frequency is important at R265H to detect an effect on the risk of gout. It is also notable that the situation is reversed for the *SLC2A9 *intronic variants (*rs11942223 *and surrogates), with a strong association in Caucasian [5-7,9,10] and Polynesian populations (driven by protective haplotypes) [11], but not in Melanesian and Asian populations, studied thus far [12,13].

In Caucasians, in addition to R265H, there are other nonsynonymous genetic variants within *SLC2A9*, including A17T (*rs6820230*), G25R (*rs2276961*), V253I (*rs16890979*) and P321L (*rs2280205*) [7]. Only *rs16890979 *is associated with gout in Caucasians [7,11], possibly because of linkage disequilibrium with the more strongly associated intronic variants that include *rs6855911 *and *rs11942223 *[7,11]. *Rs16890979 *is also associated with gout in Polynesians; however, this is also likely to be secondary to an association with the intronic variants [11]. The minor allele of *rs16890979 *is rare in Han Chinese and Japanese [12,13]. Of the other variants, only P321L has been tested for association with gout in an Asia-Pacific region population (Japanese), in whom there was no evidence for association [13]. The impact of these nonsynonymous variants on the function of SLC2A9 has not been reported to date.

Given the heterogeneity evident in risk conferred for gout at *SLC2A9 *and *ABCG2 *in the Asian and Austronesian populations studied so far, further investigation of these genes in samples derived from Asian and Austronesian people is warranted. These populations have shared geographical history, although it is increasingly evident that considerable genetic heterogeneity exists within Asians and Austronesians, at least in gout and possibly in other complex diseases.

## Conclusions

We were unable to replicate association of the *rs3733591 *variant with gout in NZ Eastern Polynesian, Western Polynesian or Caucasian sample sets. It is possible that *rs3733591 *has a weak effect in these populations; however, our study's power was limited to detecting an effect less than that previously reported (OR = 1.4). However, consistent with a previous study in Han Chinese and Solomon Islanders, our data suggest that *rs3733591 *could be a marker of severe gout in some populations.

## Abbreviations

ABCG2: ATP-binding cassette-dependent transporter G2; ARIC: Atherosclerosis Risk in Communities; FHS: Framingham Heart Study; MSU: monosodium urate; NHLBI: National Heart, Lung and Blood Institute; NZ: New Zealand; OR: odds ratio; *P*: probability value; SHARe: SNP Health Association Resource; SLC2A9: solute carrier family 2, member 9; SNP: single-nucleotide polymorphism; URAT1: urate transporter 1.

## Competing interests

The authors declare that they have no competing interests.

## Authors' contributions

JEHM and TRM helped to design the study, oversaw its execution and prepared the manuscript. PJG, AAH, JH, PBBJ, LKS and ND helped to provide clinical recruitment and prepared the manuscript. All authors read and approved the final manuscript.
